# LeukmiR: a database for miRNAs and their targets in acute lymphoblastic leukemia

**DOI:** 10.1093/database/baz151

**Published:** 2020-03-04

**Authors:** Abdul Rawoof, Guruprasadh Swaminathan, Shrish Tiwari, Rekha A Nair, Lekha Dinesh Kumar

**Affiliations:** 1 Cancer Biology, CSIR-Centre for Cellular and Molecular Biology, (CCMB), Uppal Road, Hyderabad, 500007, India; 2 Bioinformatics, CSIR-Centre for Cellular and Molecular Biology, (CCMB), Uppal Road, Hyderabad, 500007, India; 3 Department of Pathology, Regional Cancer Centre (RCC), Medical College Campus, Trivandrum, 695011, India

## Abstract

Acute lymphoblastic leukemia (ALL) is one of the most common hematological malignancies in children. Recent studies suggest the involvement of multiple microRNAs in the tumorigenesis of various leukemias. However, until now, no comprehensive database exists for miRNAs and their cognate target genes involved specifically in ALL. Therefore, we developed ‘LeukmiR’ a dynamic database comprising *in silico* predicted microRNAs, and experimentally validated miRNAs along with the target genes they regulate in mouse and human. LeukmiR is a user-friendly platform with search strings for ALL-associated microRNAs, their sequences, description of target genes, their location on the chromosomes and the corresponding deregulated signaling pathways. For the user query, different search modules exist where either quick search can be carried out using any fuzzy term or by providing exact terms in specific modules. All entries for both human and mouse genomes can be retrieved through multiple options such as miRNA ID, their accession number, sequence, target genes, Ensemble-ID or Entrez-ID. User can also access miRNA: mRNA interaction networks in different signaling pathways, the genomic location of the targeted regions such as 3′UTR, 5′UTR and exons with their gene ontology and disease ontology information in both human and mouse systems. Herein, we also report 51 novel microRNAs which are not described earlier for ALL. Thus, LeukmiR database will be a valuable source of information for researchers to understand and investigate miRNAs and their targets with diagnostic and therapeutic potential in ALL.

Database URL: http://tdb.ccmb.res.in/LeukmiR/

## Introduction

Acute lymphoblastic leukemia (ALL) is one of the most common childhood malignancies exhibiting different molecular subtypes (1). ALL is a complex blood disorder characterized by various underlying genetic abnormalities that block B or T cell differentiation and leads to abnormal cell proliferation (2). MicroRNAs are small endogenous non-coding RNAs of 18–24 nt long which can regulate gene expression either at transcriptional or post-transcriptional level (3–5). Presence of miRNA genes in the genome and their expression is crucial in regulating various biological processes including cell cycle (6), cell proliferation (7), development (8), differentiation (9, 10), hematopoiesis and apoptosis (11–13). Recent studies suggested that almost 50% of miR genes are generally located in cancer-associated genomic regions (14) and nearly 60% of human mRNAs are conserved targets that could be regulated by diverse populations of miRNAs (15). The discovery of these small non-coding RNAs as the major regulators of gene expression has revolutionized the clinical research giving new impetus for creation and optimization of novel therapeutic treatments. MicroRNA target identification is crucial for elucidating the functions of microRNAs in different oncogenic signaling pathways, while miRNA target prediction in vertebrates is more complex due to its imperfect homology at the seed region of target sequence; it is relatively easy in case of plants due to perfect or nearly perfect complementarity to their target sequences (16, 17). There are many concrete evidences of involvement of microRNAs in the development of different types of leukemias. The direct and indirect role of microRNAs in cancer has been well established by several studies in early hematopoietic differentiation (18, 19). Accumulating evidences have shown that microRNAs have been used in the classification of cancers including leukemias (20). Computational identification of miRNA targets aids in experimental validation which can fortify our understanding about microRNAs as key regulators in the development and progression of cancers. With increasing number of microRNAs discovered, it is imperative to keep a track of miRNAs published as well as define the underlying network of the miRNAs. Hence, there is a need for a systemic and dynamic repository having all miRNAs and their targets with a user-friendly interface for querying, archiving and retrieving desired information.

To date, several databases have been developed to catalogue the heteromorphic information of miRNAs, their target mRNAs and their association with diseases such as cancer. Such databases like miRBase (21), miRGator (22) and miRGen 2.0 (23) collectively provide a complete repository of miRNA nomenclature, sequence annotation and target information available in the genomes of different species. Numerous manually curated databases such as microRNAs.org (24), miRTarbase (25), miRecords (26), miRWalk 2.0 (27) and miRNAMap (28) give insight into the experimentally validated miRNAs, targets and their interactions. TargetScan (29), PicTar (30), MiRCosm and miRDB (31) are databases that contain computationally predicted miRNA-target related information in diverse species and diseases. Realizing the importance of microRNAs in diseases including cancer, some specific databases like miRCancer (32), miR2Disease (33), OncomiRdbB (34), HMDD v2.0 (Human microRNAs and disease database; (35)), S-MED (Sarcoma microRNAs Expression Database; (36)) and PhenomiR 2.0 (37) that exclusively collate information about association of microRNAs and their targets in different cancers and other diseases have been developed.

Recent expression profiling studies suggested microRNAs/mRNAs as one of the major players involved in classification of ALL from other leukemias which can further aid in assessing the clinical outcome of the disease (38–40). Hitherto, deregulation of ALL oncogenes as well as oncomiRs in various signaling pathways have been established (41). Recently, differential expression of microRNAs between infant and childhood T-ALL cases was reported (42). However, there are no databases that provide insights into miRNA targets, miRNA–mRNA interaction networks and target gene ontologies in different signaling pathways for acute lymphoblastic leukemia. Therefore, we sought to design and develop a web interface-based user-friendly comprehensive database known as ‘LeukmiR’ (www.tdb.ccmb.res.in/LeukmiR). This database contains a comprehensive information on computationally predicted acute lymphoblastic leukemias (ALLs) associated miRNAs mined from different databases, their cognate target mRNAs and the interaction network in different signaling pathways along with experimentally validated microRNAs in ALL. Herein, we present an inclusive database consisting of 861 miRNAs and 550 genes as miRNA targets in human and 656 miRNAs and 523 genes as miRNA targets in the mouse genome across various signaling pathways. Out of 861 identified human miRNAs, 51 miRNAs have not been previously reported to be associated with ALL. Moreover, the identified putative targets for microRNAs were cross validated using TargetScan and PicTar. Being an exclusive database for ALL and freely available to all, LeukmiR will be a valuable source of information that could help researchers identify microRNAs and their oncogenic targets offering promise for changing the clinical paradigm in ALL.

**Figure 1:**
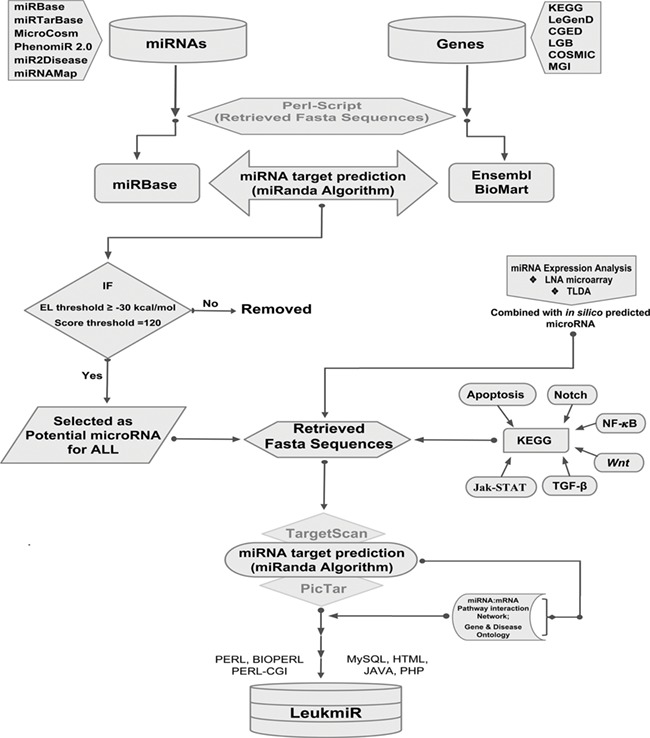
**Schematic representation of database structure and construction**. The work flow represents the mining of miRNAs and their target genes using Perl script and further incorporation into the database using mysql, perl-cgi, java, php and html.

## Database Design and Structure

### Mining and cataloguing miRNAs with their targets

All previously reported microRNAs for both human and mouse genomes were retrieved from various databases such as miRbase, MicroCosm, miR2Disease and PhenomiR 2.0. Additionally, experimentally validated microRNAs in 50 ALL patient samples by TaqMan Low Density Array (TLDA) and locked nucleic acid (LNA) array were included. Duplicate miRNAs were filtered out, and FASTA sequences of all remaining mature miRNAs were retrieved from miRBase using an in-house perl-script program. The genomic sequences (i.e. 3’UTRs, 5’UTRs and exons) of the reported ALL oncogenes catalogued in different databases such as KEGG (43), COSMIC (44), LeGenD (Leukemia Gene Database; https://www.bioinformatics.org/legend/legend.htm), LGB (LeuGenBase; http://lgb.adibiosolutions.com/), CGED (45) and MGD (46) were downloaded in FASTA format using Ensembl-BioMart tool. A total of 2568 human and 1907 mouse miRNAs were screened against the ALL oncogenes of human and mouse, respectively, using miRanda (47). This tool identifies potential miRNA target sites in genomic sequences using a local sequence alignment approach where scores are based on sequence complementarity and not sequence identity. After screening of miRNAs, KEGG database was used to identify their putative targets in different signaling pathways such as TGFβ, Apoptotic, Notch, NF-κB, Wnt and JAK-STAT. A network depicting the miRNA-mRNA interactions was constructed based on its target identification. The overall workflow representing the collection and processing of data throughout database construction is illustrated in (Figure 1).

**Figure 2:**
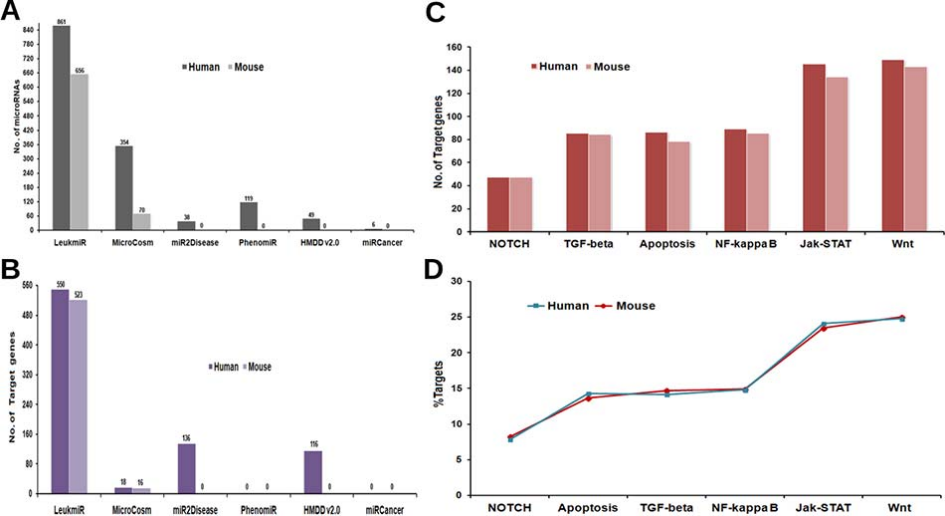
**Contents of ‘LeukmiR’**. Total number of (**A**) ALL miRNAs and (**B**) target genes for both human and mouse in LeukmiR compared to MirCosm, miR2Disease, PhenomiR, HMDD v2.0 and miRCancer databases. (**C**) Total number of microRNA targets with respect to different pathways. (**D**) Percentage of the same available in different signaling pathways.

### Database structure and interface

The LeukmiR database has been designed as an interactive web interface where the user can browse and extract information about miRNA and their targets from both human and mouse genomes. PHP and Perl-CGI script query system with a backend MySQL database running on Apache web server has been developed to represent the output of a query as a user-friendly web page generated in HTML using Java script to represent the results. MicroRNAs and their putative target information were imported into a MySQL database for creating a web interface. Search, query and data extraction system was developed using PHP and Perl-CGI scripts for searching microRNAs and their target information independently (Figure 1). The database also links users’ query to the original databases for detailed information. Thus ‘LeukmiR’ facilitates the user to access the particulars about ALL microRNAs, their accession numbers, putative targets and chromosome location. It is a freely accessible database which is hosted on the web and allows users to access information about the microRNAs and their putative targets exclusively for ALL.

## Construction and Content

### Classification of microRNAs as potential ALL miRNAs

For classification of miRNAs as putative ALL miRNAs, target prediction was performed against the ALL oncogenes using miRanda with pairing score threshold of 120 at different energy levels (i.e. EL −15, −20, −25 and −30 kcal/mol). Those microRNAs predicted to have ALL oncogenes as targets at the most stringent energy level (EL ≤ −30 kcal/mol), and their sequence complementarity to the target genes were classified as ALL microRNAs. Based on these parameters, we identified and classified 861 human and 656 mouse miRNAs as potential microRNAs associated with ALL from existing databases. The utility of integrating various databases with additional new information into a single comprehensive database could be envisaged by a comparison between LeukmiR database and other existing databases (Figure 2A). MicroRNAs that did not meet the criteria were filtered out from further studies. These predicted miRNAs were then cross validated with two different array-based expression profiling techniques/platforms using blood/bone-marrow samples of ALL patients.

### Identification and validation of novel miRNAs using expression profiling

TaqMan Low Density Arrays (TLDA version 2.0) containing 667 human microRNAs in two array plates Pool A and Pool B, covering Sanger miRBase version 10, was used to validate microRNA expression profiling in 50 ALL patient samples. (This study was approved by Institutional review board and ethics committee of RCC and Centre for Cellular and Molecular Biology (CCMB) and all procedures were performed following the declaration of Helsinki, after obtaining written informed consent from the parents/guardians.) A pre-amplification step of cDNA with PreAmp Megaplex pool primers was done to ensure and enhance the detection of miRNAs expressed at low levels in the samples. One TaqMan microRNA assay, not related to human, was used as negative control with RNU 46 and RNU 48 as endogenous controls. PCR reactions, preceded by reverse transcription, was carried out in ABI 7900HT. The data was normalized using endogenous controls, and all statistical analyses were performed using Spotfire (StatMiner) software. The TLDA data set is available at Gene Expression Omnibus (GEO: http://www.ncbi.nlm.nih.gov/geo/) with accession number GSE60386.

The same experiment was repeated and validated by exiqon’s LNA arrays (locked nucleic acid; Array version v11.0) which contained 1372 microRNAs (hmr-miRBase 14.0 + miRPlus) by swapping of the dyes. LNA array slides were scanned using Agilent G2565BA microarray scanner system (Agilent Technologies, Inc. USA) and further image analysis was done using ImaGene 8.0 software (BioDiscovery, Inc., USA). Quantified signals were further background corrected and normalized using global Lowess (Locally Weighted Scatterplot Smoothing) regression algorithm (48). The complete data set for LNA arrays is available at GEO with accession number GSE59199.

Experimental validation on human ALL samples identified 397 valid and significant microRNAs from both array platforms together. A total of 193 (~49%) out of 397 microRNAs were matched with *in silico* mined/predicted microRNAs. Fifty-one of these were classified as novel ALL miRNAs as they were not reported to be associated with ALL earlier. Targets for novel microRNAs in different signaling pathways were identified using miRanda. Duplicate entries were removed, and experimentally validated ALL miRNAs were merged with *in silico* predicted microRNAs using in-house perl-script. Target prediction for these microRNAs to the genes involved in different signaling pathways was done using the miRanda program (Supplementary Table 1).

### Target identification and miRNA–mRNA interaction network enrichment in KEGG pathways

Regulation of both spatial and temporal expression by different signaling cascades through miRNAs has been reported to play a significant role in ALL (49–54). Hence, plausible target genes of the predicted ALL miRNAs from different signaling pathways such as TGFβ, Apoptotic, Notch, NF-κB, Wnt and JAK-STAT from KEGG were identified using the miRanda algorithm at four different energy levels with score threshold value 120. In the subsequent steps, TargetScan and PicTar were additionally used to validate the predicted targets. A total of 550 and 523 genes as miRNA targets collectively from all signaling pathways were identified for human and mouse, respectively. The number of genes as targets for ALL specific microRNAs is higher in LeukmiR compared to other cancer databases such as MirCosm, miR2Disease, PhenomiR, HMDD v2.0 and miRCancer database (Figure 2B). JAK-STAT and Wnt signaling pathways recorded the highest number of genes targeted by ALL miRNAs, while Notch signaling had the least number of targets among all signaling pathways (Figure 2C and D).

It is already known that a single miRNA can target one or multiple mRNAs or a single mRNA can be targeted by multiple miRNAs. Therefore, it would be appealing to study their interaction in signaling pathways, which could provide better perception about the possible miRNA-directed regulation. Considering the crucial regulation of mRNAs by microRNAs in disease development, we have used the information on miRNAs and their targets in each signaling pathway to identify their interaction. A binary matrix was created for each signaling pathway to depict miRNA and target interaction in terms of 1 and 0 (1 = mRNA targeted by miRNA; 0 = not targeted). As a result, a network depicting the interaction between miRNAs and their putative targets (Supplementary Figure S1) was generated through *gplot* function of *sna* package (55) in open source R (v 3.10) (https://www.r-project.org/).

**Figure 3:**
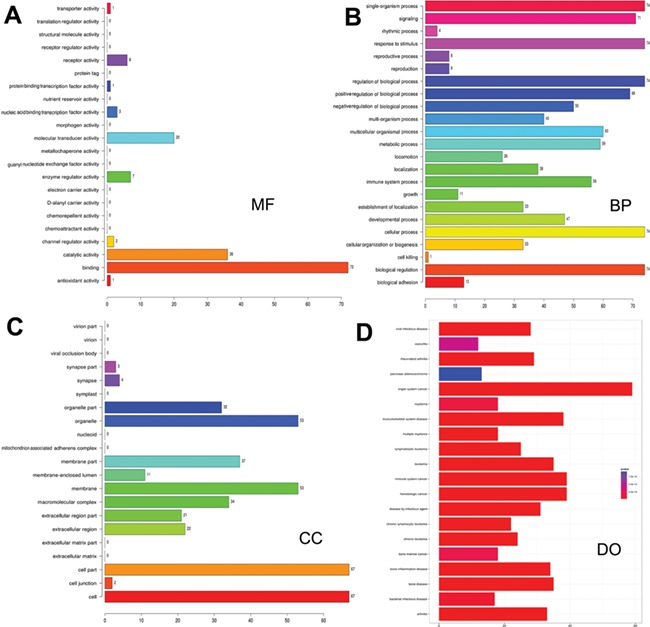
**Gene ontology and disease ontology of miRNA targets in various signaling pathways**. The gene ontology of targets represented in terms of their associated (**A**) molecular function (MF), (**B**) biological process (BP), (**C**) cellular component (CC) and (**D**) disease ontology of target genes.

### Functional annotation of microRNA targets and disease ontologies

As many miRNAs are being identified and their targets predicted, there is a paradigm shift in research to infer the functions of the predicted miRNAs and their targets. Hence, functional enrichment analysis of miRNA targets in different signaling pathways was performed using Gene Ontology (GO) database, and the association of these target mRNAs with various diseases was assessed. Functional annotations for targets were identified by using R/Bioconductor packages *goProfiles,* which performs functional profiling of a list of genes based on their projection at a given level of Gene Ontology (56) and *DOSE* (57). The LeukmiR database provides gene ontology of miRNA targets in oncogenic pathways in terms of their associated biological processes (BP), cellular components (CC) and molecular functions (MF) in a species-independent manner along with their disease ontology information (Figure 3). Here, BP relates to a series of biological events of a gene product with a defined outcome, while MF and CC describe activities of a gene product at molecular level and location, respectively. This contains plausible miRNA targets of different signaling pathways, which could further provide insight into the common molecular mechanisms underlying diseases regulated by microRNAs.

### User interface and database accessibility

LeukmiR database is an *in silico* platform with user-friendly search options to retrieve meaningful information of ALL oncomiRs from two different genomes (human and mouse). This includes their mature sequences, respective target sites in different oncogenic pathways and location in their respective chromosomes. MicroRNAs with their respective target interaction network in various signaling pathways are also presented along with useful ontology (Gene Ontology and Disease Ontology) information of target genes.

On the search page, the user can access and retrieve miRNA and their target information in three ways. A general search bar is available on the left bottom side with the caption ‘Search LeukmiRdb’. This search box allows for text-based search using miRNA name, its accession number, sequence, gene name, Entrez ID, chromosome name (‘chr7’ or ‘Chr7’ or Chrn where *n* is the number of any chromosome X and Y) or pathway name as query input. Quick search is case insensitive and accepts any fuzzy term (minimum size of three characters) related to miRNA, target gene or signaling pathways as input to search whole database. More specific options are provided by applying filters depending on the user’s choice. The user can narrow the search by selecting the species (human or mouse) and using ‘miRNA identifier’ which is specific, where the user has to provide exact miRNA name, its miRBase ID, its sequence, accession number or mature sequence. Search by ‘target gene/mRNA identifier’ is also a specific search-based query method where the user can input gene name, ensembl ID and entrez ID irrespective of their case to check the availability of mRNA in the database. In this case, the user needs to enter the complete name/accession number of the miRNA/mRNA.

The result page displays the results in a table representing each microRNA and its respective target information in separate columns. The table also displays microRNA target sites (3′UTR, 5′UTR or Exon) at four different energy levels (EL: −15, −20, −25 and −30 kcal/mol) with their associated signaling pathway information. The first two columns, ‘miRNA’ and ‘accession’, provide a direct link for miRNA annotation and FASTA sequences in miRBase (www.mirbase.org). The next column ‘Target_Gene’ directs target gene information to GenAtlas (www.genatlas.org) in terms of gene structure, function, expression and mutations (58). The ‘Ensembl_ID’ column has a link to the Ensembl database (www.ensembl.org) which has gene summary, its annotation and transcript information. The ‘Entrez_ID’ column also has similar information in a customizable gene report layout by directing gene information via entrez ID to the BioGPS (www.biogps.org) portal (59). The column ‘Target_Site’ indicates the predicted miRNA site on the target gene which could be 5′UTR, 3′UTR or exonic regions of the gene. The ‘Signaling_pathway’ column gives information about the signaling pathways obtained from KEGG. The ‘Chromosome’ column as the name suggests includes chromosomal location of target genes by directing its link to GeneCards (http://www.genecards.org) which has a wide range of gene-centric information (60). The last column of result table represents different energy levels at which the miRNA targets were predicted using the miRanda program. Users can also sort query result by clicking on the table header columns with miRNA name, target gene, chromosome or energy level. Overall, the result window gives comprehensive information of all ALL microRNAs and their putative targets at different energy levels.

On the same search page, the user can also interrogate the miRNA:mRNA interaction network in different signaling pathways for selected species. The result page for a interaction network in a particular KEGG pathway represents miRNA and target relation at three different genomic locations such as 3′UTR, 5′UTR and Exon. The user can zoom into the network by moving its cursor on the image to get details on direct or indirect miRNA targeting of the gene in the signaling pathway. The interaction result page also directs a link (new page) for gene ontology (GO) and disease ontology (DO) profiles of genes predicted as miRNA target. This section also facilitates users to obtain experimentally validated ALL microRNAs along with their expression values.

## Results and Discussion

Though over the years, a number of databases for microRNAs and their targets have been created, only a few of them have catalogued miRNAs and their involvement in specific diseases in human. Till date, there is no database available with information exclusively on the association of microRNAs with ALL. Herein, we present a dynamic and user-friendly database named ‘*LeukmiR*’ with comprehensive information of *in silico* predicted and experimentally validated microRNAs with their putative targets. This could further facilitate identification of potential biomarkers for ALL with diagnostics and therapeutic features. Compared to other databases like MicroCosm, miR2Disease, PhenomiR, HMDD v2.0 and miRCancer, this database contains maximum number of ALL specific microRNAs with 861 in human and 656 in mouse (Figure 2A). Out of 397 experimentally validated miRNAs, 51 were not reported earlier to be associated with ALL. These novel microRNAs with their target information in different pathways are represented in Supplementary Table 1. The LeukmiR database not only has a user-friendly web interface to query miRNAs, target mRNAs and their interaction network but also provides target-ontology information for all genes in oncogenic pathways. It is a widespread platform for studying the miRNA and their plausible role in development, and progression of ALL.

Identification of microRNA targets and its functional prediction is a vital factor in assessing the role of microRNAs in regulating/deregulating common genes and pathways responsible for development of cancer (61). Though algorithms for miRNA target prediction are continuously evolving, effective prediction of miRNA targets in animals is a more complex task than in plants (62). MicroRNA regulates target genes via binding at 3′UTR/5′UTR or coding region of genes through seed region (5′ end of miRNA) making it a more challenging task for target prediction tools by incomplete base pairing at the complementary site (63). Here, we applied a well-known target prediction algorithm miRanda at four different energy levels with validation by PicTar and TargetScan for minimizing false positives. Targets predicted at lowest energy level (EL −30) represent strong pairing of miRNA to its target, whereas EL −15 is a more relaxed criterion (used for finding novel miRNA targets). This will help researchers to narrow down and screen most favorable putative microRNA targets in ALL for validation. The LeukmiR database consists of 550 and 523 miRNA targets for human and mouse genome, respectively, which is the highest number of predicted putative targets for ALL miRNAs compared to any other available database (Figure 2B).

Negative regulation of diverse oncogenes through microRNAs in different signaling pathways maintains a healthy environment in normal cells. Their subsequent deregulation could be a crucial mechanism to understanding the initiation and progression of cancers. Therefore, classifying ALL miRNA targets based on different signaling pathways and deciphering miRNA:mRNA interaction network at 3′UTR, 5′UTR and exon regions of target genes could provide detailed information on the role of miRNAs in ALL. Cross talk between miRNA and signaling pathways during hematopoiesis, cell proliferation and differentiation in ALL has been reported in several studies. Wang *et al*. (2010) reported cross talk between miRNA and NOTCH signaling in different cancers including acute lymphoblastic leukemia (64). Chan *et al*. (2007) described the impact of regulation of the mTOR pathway through Notch signaling on leukemogenesis and cell growth in T-ALL (65). The LeukmiR database facilitates the mining of information on such cross talks through the miRNA:mRNA interaction network in different signaling pathways. For instance, miR-150-5p was predicted to target SMAD4 and NOTCH3 in the TGF-β and NOTCH signaling pathways, respectively. Also, miR-150 acts as a key tumor-suppressor gene and has been reported to have low levels in various types of hematopoietic malignancies including leukemias (66). Moreover, Ghisi *et al*. (2011) identified the adverse effect of forced expression of miR-150 on T-cell proliferation and survival by downregulating NOTCH3 expression in NOTCH signaling (67). Further, SMAD4, which is a core component of the TGF-beta signaling pathway, has been reported as a growth inhibitor of human hematopoietic progenitors by Blank *et al* (68). In the LeukmiR database, both NOTCH3 and SMAD4 were predicted targets for miR-150, and with such prior information, the plausible role of miR-150 could be hypothesized in regulating hematopoiesis and cell proliferation through interacting with NOTCH and TGF-beta signaling pathways.

Though deregulation of microRNAs has been linked with many cancer types including hematological malignancies, very little is known about their causative effects on pathway regulation. In pediatric ALL, tumor-suppressor microRNAs miR-100 and let-7e were reported to be down-regulated while miR-128a and miR-181b were found to be overexpressed in bone marrow samples as compared to normal samples (69). In the LeukmiR database, these down- and up-regulated microRNAs were commonly found to target Wnt and JAK-STAT pathways (Figure 2C). In the present database, both miR-100 and let-7e were also predicted to target SMAD2, a tumor suppressor gene overexpressed in many cancers (70). Likewise, miR-128a and miR-181b were shown to target SMURF1, ACVR1C, INHBA, STAT1, JAK1 and SOS1 in TGF-beta and JAK-STAT signaling pathways. Further, Xioa *et al*. have demonstrated the overexpression of miR17–92 cluster comprising of six miRNAs (miR-17, miR-18a, miR-19a, miR-20a, miR-19b-1 and miR-92-1) during B and T-cell proliferation, hematopoiesis in ALL and other cancers (71). In our database also, the miR-17-92 cluster has been predicted to target a diverse number of genes such as PRKCB, BCL2A1, LMO2, ACVR1, THBS4, TGFB1, SMURF2, CBLB, STAT5B, MYC, IKBKB, XIAP from NF-kB, TGF-beta, JAK-STAT and Wnt signaling (Figure 2D). This information could help in further understanding the role of the miR-17-92 cluster in regulation/deregulation of the intricate cross talk during cell proliferation, maturation and apoptosis during abnormal hematopoiesis or leukemogenesis. With such prior information and knowledge of the putative targets, it could provide a platform for understanding these microRNAs in regulation of diverse pathways leading to formulation of novel therapeutic regimens circumventing ALL.

Increasing evidence indicates that functional annotation of miRNA targets at different cellular and molecular levels are very important for systematic ontology of microRNAs. Assigning ontology to targets of microRNAs is another feature of this database keeping in view of their association with biological processes at cellular and molecular level. Such information could also decipher the possible role of microRNAs in a series of molecular or biological events of target genes, thus providing ontology function to the miRNAs. Moreover, exploring relations between miRNA target genes and diseases could simplify our understanding towards pathogenesis of cancer and its progression (Figure 3).Though our study has placed a plethora of information for the scientific community, further extensive experimental validation is required to bring about these molecules as functional biomarkers for diagnosis, prognosis and therapeutic molecules for acute lymophoblastic leukemia.

Another class of non-coding RNAs known as the long non-coding RNAs (lnc RNAs) has emerged as a novel class of molecules involved in the pathophysiology of hematological malignancies. Recently, some of them have been identified that are associated with relapse specificity in B-ALL (72). The understanding of lncRNA biology and leukemia is now at intersections where more studies are required to ascertain their role in the development and progression of the disease. Therefore, the scope of LeukmiR can be widened to incorporate such functionally defined systems in future that may further help in unifying mechanistic themes in ALL.

## Conclusion

‘LeukmiR’ is a miRNA-target database which could bridge the gap among publically available databases by providing microRNAs and their target information exclusively for acute lymphoblastic leukemia (ALL). ‘LeukmiR’ is an all-inclusive database where the user can acquire consolidated information on computationally predicted as well as 397 experimentally validated ALL microRNAs and their targets. This includes a set of 51 novel microRNAs that are not reported or classified earlier as ALL-specific microRNAs. Moreover, LeukmiR also provides information about microRNA-regulated signaling pathways through a miRNA:mRNA interaction network that could substantially reduce time-consuming efforts in searching, and identification of appropriate targets for validation. In order to maintain the applicability and dynamics of the database, it will be updated in a yearly manner.

## Availability of database

This database is freely accessible at http://tdb.ccmb.res.in/LeukmiR/ without any academic or non-academic restrictions.

## Supplementary Material

Figure_S1_baz151

Supplementary_Table1_baz151
