# Supplementary figures and images for: LeukmiR: a database for miRNAs and their targets in acute lymphoblastic leukemia
Source: Database (Oxford). 2020 Mar 4;2020:baz151. doi: 10.1093/database/baz151 (PMC7054207; doi:10.1093/database/baz151)

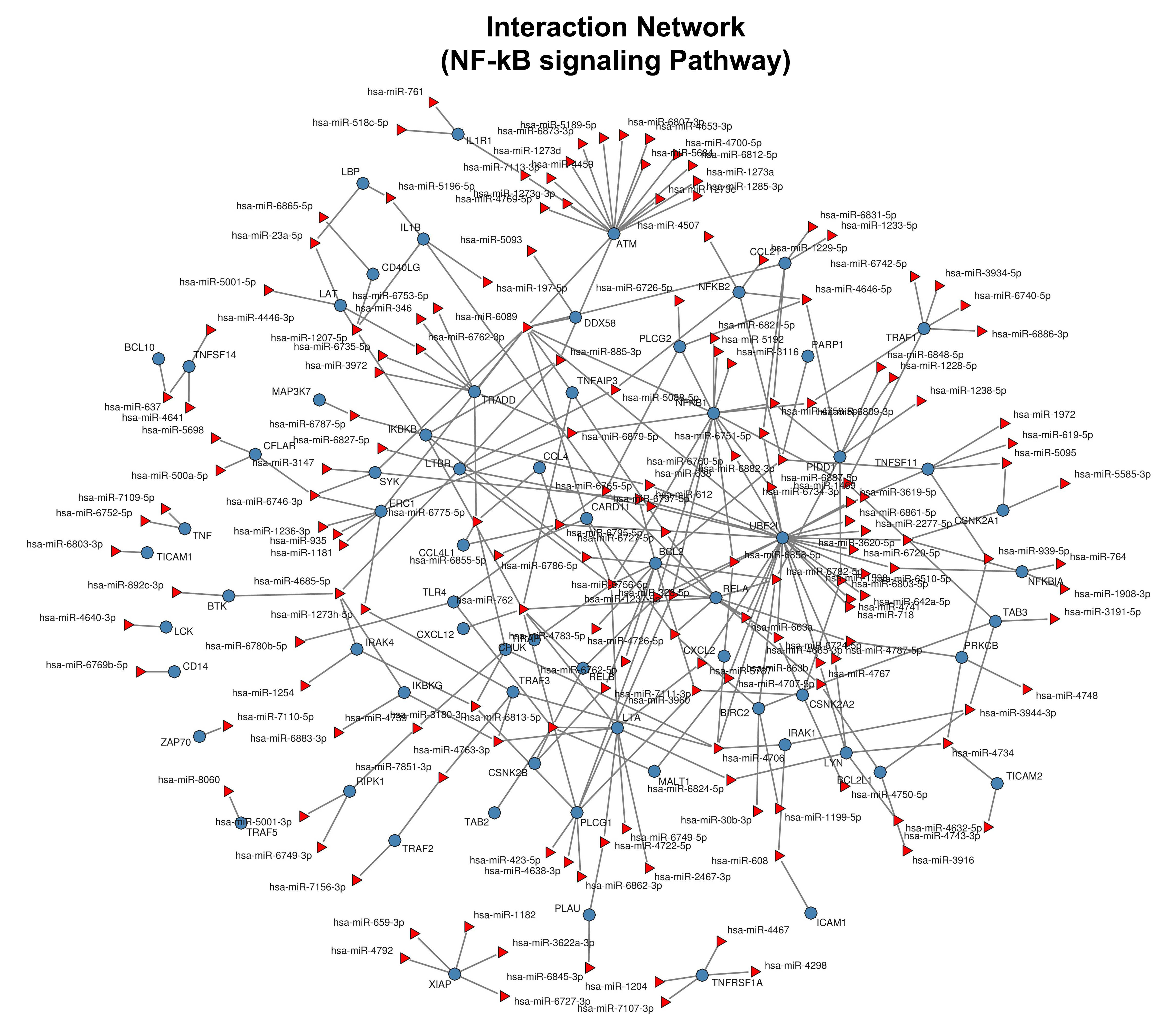

Supplement: Figure_S1_baz151 [file figure_s1_baz151.png]
